# The Impact of Predation Risks on the Development and Fecundity of *Bactrocera dorsalis* Hendel

**DOI:** 10.3390/insects15050322

**Published:** 2024-05-01

**Authors:** Xin Liu, Jian Wen, Xingyu Geng, Lu Xiao, Yan Zou, Zhe Shan, Xianli Lu, Ying Fu, Yu Fu, Fengqin Cao

**Affiliations:** School of Tropical Agriculture and Forestry, Hainan University, Haikou 570228, China; 22220951320066@hainanu.edu.cn (X.L.); 996095@hainanu.edu.cn (J.W.); 22210904000013@hainanu.edu.cn (X.G.); 22220951320032@hainanu.edu.cn (L.X.); 21210904000032@hainanu.edu.cn (Y.Z.); 21220951320083@hainanu.edu.cn (Z.S.); 23220951320098@hainanu.edu.cn (X.L.); 23220951320055@hainanu.edu.cn (Y.F.); 23220951320069@hainanu.edu.cn (Y.F.)

**Keywords:** *Bactrocera dorsalis* Hendel, non-lethal effects, phenotypic plasticity, predator–prey interactions, pest management

## Abstract

**Simple Summary:**

The oriental fruit fly, *Bactrocera dorsalis*, negatively impacts global agriculture. One promising mitigation strategy is biological control, emphasizing a comprehensive understanding of predator influences: specifically, the effects of predation risk on *B. dorsalis* development and reproduction. Our study investigated the predation risk effects of the mantis *Hierodula patellifera* Serville on *B. dorsalis*. We found that exposure to this predator or its odor shortened developmental time and increased fecundity in female *B. dorsalis*. However, male development and the death weights for both sexes remained stable. Our findings extend the understanding of *H. patellifera*’s predation risk effects on *B. dorsalis*’ life history, shedding light on potential alterations in the fitness and population dynamics of *B. dorsalis*. Additionally, we identified the use of predator odor as a possible innovative method for controlling *B. dorsalis*.

**Abstract:**

Predators are dependent on the capture of prey to meet their energetic and nutritive requirements, which brings the risk of predation to prey. The predation risk is divided into consumptive and non-consumptive effects. Non-consumptive effects may manifest through altered growth and ontogenetic trajectories in prey species, a dynamic modulated by olfactory or other sensory cues from predators. *Bactrocera dorsalis* Hendel represents a major invasive threat to global horticulture. While earlier research was primarily centered on the consumptive interactions between *B. dorsalis* and its natural enemies, the potential consequences of non-consumptive interactions on the development of *B. dorsalis* have been overlooked. In this study, we investigated the impact of predation risk effects, induced by both visual exposure to the predatory mantis *Hierodula patellifera* Serville and its associated odor, on the life history traits of *B. dorsalis*. Female *B. dorsalis* demonstrated a reduced developmental time in the presence of a caged predator (*H. patellifera)* or predator odors, but showed significantly increased fecundity. Conversely, males displayed no significant change in developmental time. Additionally, neither the female nor male body weight at death was significantly influenced by the predation risk from the caged predator or predator odors. This study investigated the effects of predation risk on the development and reproduction of *B. dorsalis*, emphasizing the potential importance of odor risk in biological and pest control.

## 1. Introduction

The interaction between predators and prey is a fundamental form of biological interaction [1,2]. Predators depend on prey to obtain energy and nutrients, while prey are subject to pressure and control from predators [3]. The interactions between predators and prey can be divided into two main types: consumptive and non-consumptive effects [4,5]. Consumptive effects reduce prey density through lethal predation, while non-consumptive effects affect prey populations by influencing prey fitness [6,7,8,9,10]. Analyses have shown that the non-consumptive effects of predators on prey may exceed the impact of consumptive effects on prey populations [3,11]. However, the research on non-consumptive effects is limited, and there is even less research on non-consumptive effects in insects.

When organisms experience non-consumptive effects, they often exhibit a trade-off strategy [12,13]. Acquiring resources for development and avoiding predators constitute an important trade-off, with organisms allocating energy accordingly [14,15]. In the presence of predators, prey often allocate more energy to defense mechanisms against predation [7]. For example, when detecting the presence of predation stress, potato beetle larvae (*Leptinotarsa decemlineata*) reduce feeding to avoid predation [16]. Female damselflies (*Ischnura cervula*) in water exhibit a reduced feeding desire when they sense predator cues, allocating more resources to finding predator-free habitats [17].

Such defensive strategies often lead to shifts in growth rates and fecundity [18,19]. For example, butterfly larvae (*Pararge aegeria*) develop rapidly when facing predation risks because they allocate more energy resources to growth and development, despite the increased risk of immediate mortality [20]. Long-term exposure to a predation threat, such as the threat from the six-spotted ladybug (*Menochilus sexmaculatus*), significantly reduces the lifespan of adult cotton aphids (*Aphis gossypii*) [21]. In the case of the herbivorous mite (*Tyrophagus putrescentiae*), individuals in control conditions exhibit a significantly longer lifespan than those subjected to predation risk, regardless of whether the stress occurs throughout their entire life cycle or only at specific stages [22]. The duration of the predation risks also affects lifespan; adult *Drosophila melanogaster* individuals experience a significantly shorter lifespan when under continuous predation risk from ladybugs (*Propylea japonica*) compared to those facing intermittent risk [23]. Predation risk often results in a decrease in prey fecundity; for instance, *Sitobion miscanthi* reduces its net reproduction rate in response to isolated predator presence [24]. Female *Bactrocera tryoni* lay fewer eggs in the presence of predator olfactory cues [25]. However, there are instances where predation risk has no significant effect on prey fecundity, as seen in *Philetairus socius* [26].

Prey can detect predator cues in the environment through vision, hearing, smell, and touch, using this information to adjust their behavior and avoid predation [27,28]. Visual features such as the appearance, body shape, color, and movement patterns of predators help prey identify potential hazards and trigger avoidance behavior [29,30,31]. Additionally, the specific vocalizations of predators serve as a means for various prey species to assess threatening situations [32]. However, when olfactory cues are unclear or absent, prey must rely more heavily on visual judgment to assess predation risk, which requires them to come closer to the predator [33]. Prey animals themselves release or store characteristic chemical cues, which other animals detect and use to alter their behavior to reduce their predation risk [34,35,36]. An accurate assessment of predation risk enables animals to exhibit effective anti-predator behaviors. For example, *Bactrocera tryoni* significantly reduces foraging, oviposition, and mating in the presence of predator volatiles [25]. The negative reaction of *Leptinotarsa decemlineata* to male volatile cues from predators (*Podisus maculiventris*) has been shown to be particularly strong [16]. However, currently, the research on the effects of threats mediated by predator odor is limited.

The mantis, *Hierodula patellifera* Serville, is widely distributed in East Asia and Southeast Asia. It is considered an efficient predator, known for its sit-and-wait hunting strategy, and serves as an important natural enemy of *B. dorsalis* and other dipteran and lepidopteran pests in orchards [37,38]. *Bactrocera dorsalis* Hendel, commonly known as the oriental fruit fly, is a fruit pest widely distributed in tropical and subtropical regions of Asia. Since its invasion into China, it has brought considerable harm to various crops and fruits, and the scope of harm has been expanding [39,40]. At present, the use of chemical control methods has adversely affected the environment and has accelerated the development of resistance to pesticides in *B. dorsalis* [41,42,43,44]. Biological control presents a promising alternative to reduce *B. dorsalis* populations. In order to improve the effectiveness of biological control, it is necessary to understand the impact of predation risks on *B. dorsalis*, which plays a crucial role in biological control strategies.

In this study, we aimed to investigate the effects of predation risks from *H. patellifera* on the development and reproduction of *B. dorsalis*. Two types of predation risk were set: predation risk from the caged predator [45,46] and from predator odors. We predicted that both predation risks from caged *H. patellifera* and from *H. patellifera* odor could alter the development and reproduction of *B. dorsalis*, which may have major impacts on the population dynamics and biological control of *B. dorsalis*.

## 2. Methods and Materials

### 2.1. Experimental Insects

**Figure 1:**
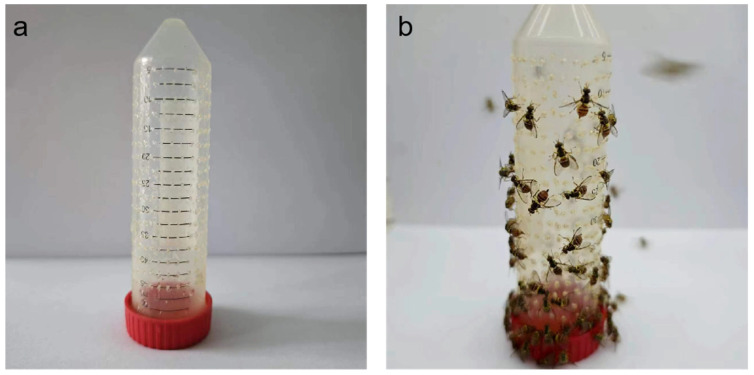
Egg-collecting tube (**a**) made of 50 mL centrifuge tube. *B. dorsalis* deposit their eggs directly into the egg-collecting tube (**b**).

Larvae of *B. dorsalis* utilized in this study were sourced from the Haidian campus of Hainan University in Haikou, China. Maintained under controlled conditions of 27 ± 1 °C temperature, 65% relative humidity, and a 16:8 h (L:D) photoperiod, the larvae were nourished on a diet comprising paper, banana, corn flour, sugar, yeast powder, concentrated hydrochloric acid, sodium benzoate, and sterile water [47]. Adults of *B. dorsalis* were housed in an insect-rearing cage measuring 80 × 80 × 80 cm, where they fed freely on artificial diets consisting of sugar, yeast, honey, agar, and water, and water was provided, and fresh food was regularly replenished. Female *B. dorsalis* were collected for fecundity using centrifuge tubes containing fresh orange juice [25] (Figure 1).

The experimental population of *H. patellifera* utilized in this study originated from a mixed clone, initiated by hatching eggs gathered from the field (Haidian campus of Hainan University, Haikou, China) and purchased from an online store (https://m.tb.cn/h.gbNMD0zEXsbuZRK?tk=ZxSOWHWXhz8, accessed on 28 April 2024.). In the laboratory, the breeding protocol for *H. patellifera* involved feeding first- and second-instar larvae with an artificial liquid diet composed of pure milk and honey, with intervals of 72 h, and subsequently providing third-instar-to-adult *H. patellifera* with a mixture of *B. dorsalis*, *Bactrocera cucurbitae*, and *Zelugodocus tau* at similar intervals, supplemented with sterile water.

Subsequently, two distinct experiments were established for investigating the predation risk effects of *H. patellifera* on the development and reproduction of *B. dorsalis*. In the first experiment, *B. dorsalis* were reared in an environment isolated from the predator *H. patellifera*, enabling detection of the predator’s presence through visual cues and odors. In the second scenario, *B. dorsalis* was exposed solely to the odors associated with *H. patellifera*, allowing detection of the predator exclusively through olfactory cues. The development time, fecundity, and weight at death were recorded.

#### 2.1.1. The Effect of Caged Predators on the Development and Fecundity of *B. dorsalis*

Adult *B. dorsalis* that emerged within a 24 h period were selected for the study. For the risk treatment, 40 *B. dorsalis* individuals were housed in a 20 × 20 × 20 cm insect-rearing cage, maintaining a 1:1 male-to-female ratio, with daily provision of diet. A seventh-instar *H. patellifera* (starved for 24 h) was placed in a caged-predator device (a transparent box (15 × 10 × 5 cm) with a mesh window (10 × 5 cm) on the lid) and positioned at the bottom of the rearing cage. This setup prevented direct physical contact or feeding interactions between *H. patellifera* and *B. dorsalis*, while allowing for the transmission of risk cues through visual and olfactory channels. Test mantises were replaced every 24 h. The control treatment consisted of an empty box without *H. patellifera.* Each treatment was replicated five times, the deceased *B. dorsalis* in the cage were taken out every day, and the developmental time of *B. dorsalis* and the body weight at death were recorded. From the 8th day (the day that females start to oviposit often) of the experiment, an egg-collection tube (Figure 1) containing 5 mL of fresh orange juice was placed into the cage from 15:00 to 18:00 every day. Eggs laid by the *B. dorsalis* were collected and counted daily until the last female *B. dorsalis* in the cage died.

#### 2.1.2. The Effect of Predator Odor on Development and Fecundity of *B. dorsalis*

The predator’s odor was obtained via *H. patellifera* remaining on moist tissue paper when they moved freely in the odor-collection plastic box (19 × 12.5 × 7.5 cm). Prior to odor collection, the plastic box was cleaned with 75% ethanol, and then moist tissue paper (13 × 9.5 cm, referred to as the ‘risk paper’) was placed at the bottom of the plastic box. A seventh-instar *H. patellifera* was introduced into the plastic box and allowed to move freely for 24 h. The ‘risk paper’ was removed and placed in a new clean plastic box to be used as the odor source of the predator for each replicate. For control treatments, a clean moist tissue paper was placed in a clean plastic box. The other materials and processes were identical to the described procedure above in the caged-predator experiment, but live mantises were replaced by ‘risk paper’ in the risk treatments, or a control paper was placed in the plastic box instead of an empty box. Test tissue papers for both risk and control treatments were replaced every 24 h. Each treatment was replicated five times, and the developmental time, fecundity, and weight at death were recorded as described previously.

### 2.2. Data Analysis

The normality and homogeneity of variance of the data were assessed using Shapiro–Wilk and Levene’s tests, respectively. Survival analysis was conducted to examine the development of *B. dorsalis* under both risk and control treatments, considering differences between sexes. A Cox proportional hazard model [48] was employed to evaluate variations in survival times, while the log-rank test was utilized to compare survival curves. Fecundity analysis involved a linear mixed model (LMM) to assess the impacts of predation risk and time on *B. dorsalis* fecundity, with time designated as the random effect. Additionally, a linear mixed model was used to analyze the effects of predation risk, sex, and time on the body weight of deceased *B. dorsalis*, with time of death considered as the random effect. Normality and variance homogeneity were confirmed using the qqPlot function from the ‘car’ package and the plot.lme function from the ‘nlme’ package, respectively. All statistical analyses were conducted in R (R Core Team, 2023).

## 3. Results

### 3.1. Developmental Time

**Figure 2:**
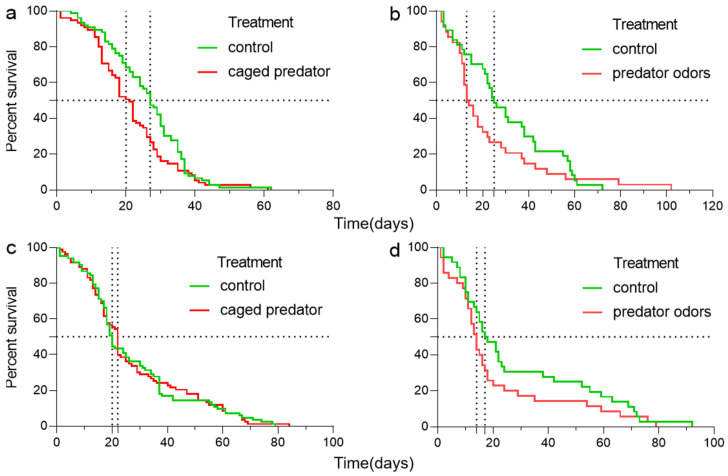
The lifespan of female (**a**,**b**) and male (**c**,**d**) *B. dorsalis* when exposed to an caged predator (**a**,**c**) and predator odors (**b**,**d**). Curves were fitted using log-rank test.

The developmental time of *B. dorsalis* was significantly affected by the predation risk from a caged predator or predator odor, but not by sex or the interaction between predation risk and sex (Table A1a,b in the Appendix A). A female *B. dorsalis* exposed to a caged predator or predator odor had a shorter developmental time (for the caged-predator experiment, the median survival time in predation risk and control treatments was 21 and 27 days, respectively; those for the predator-odor experiment were 13.5 and 25 days, respectively. Figure 2a,b). However, the developmental time for males was not affected (the median survival times in the predation risk and control treatments were 22 and 20, respectively; those for the predator-odor experiment were 14 and 17.5 days, respectively; Figure 2c,d).

### 3.2. Fecundity

**Figure 3:**
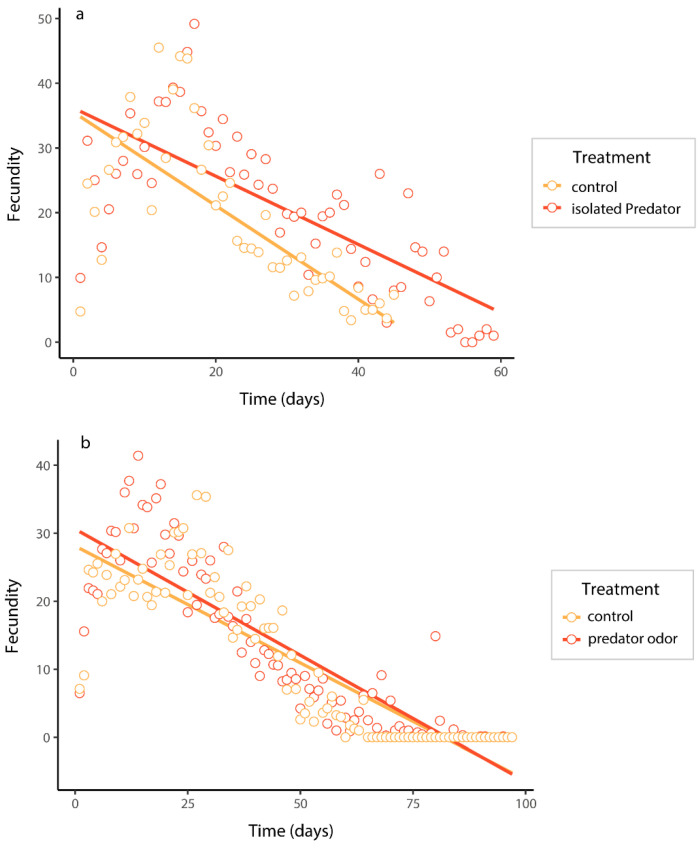
The fecundity of *B. dorsalis* when exposed to an caged predator (**a**) and predator odors (**b**). Predicted curves were fitted using linear mixed models.

In the caged-predator experiment, the fecundity of *B. dorsalis* was significantly impacted by the predation risk, time, and their interaction (Table A2a in the Appendix A). Females exposed to predation risk had higher fecundity than those in control (Figure 3a). The fecundity of *B. dorsalis* under predator-odor treatment was also significantly influenced by the same factors, but was not influenced by their interaction (Table A2b in the Appendix A). Females exposed to predator odor exhibited significantly higher fecundity than the control (Figure 3b).

### 3.3. Body Weight at Death

**Figure 4:**
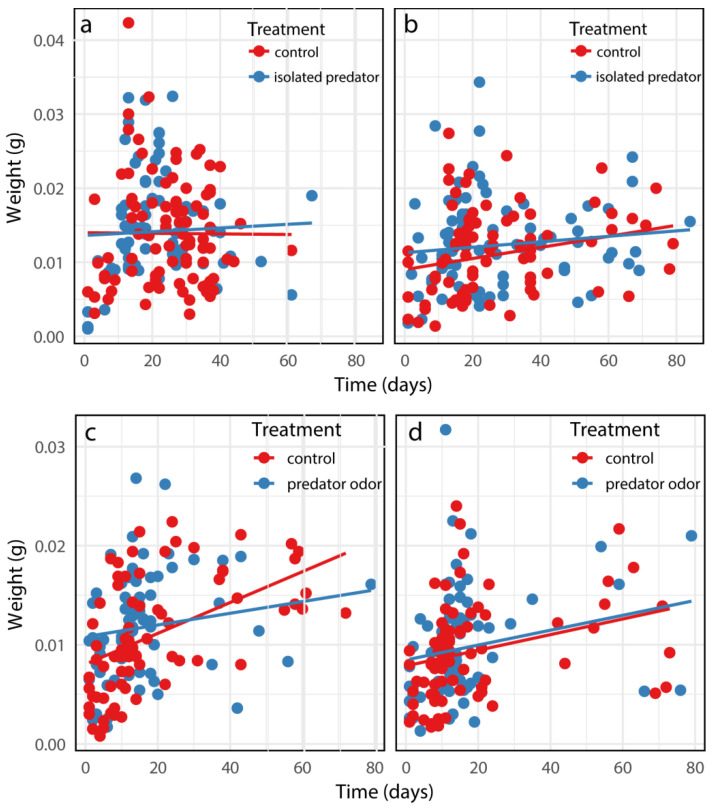
The body weight at death of female (**a**,**c**) and male (**b**,**d**) *B. dorsalis* when exposed to an caged predator (**a**,**b**) and predator odors (**c**,**d**). Predicted curves were fitted using linear mixed models.

In the caged-predator experiment, even though the gender effects on body weight at death were highly significant, no effects were observed for the time, risk treatment, or their interactions on body weight at death (Table A3a in the Appendix A; Figure 4a,b). In the predator-odor experiment, significant effects were found for gender and time, but none were observed for predation risk treatments; additionally, the interactions among treatment, time, and gender were not significant (Table A3b in the Appendix A; Figure 4c,d).

## 4. Discussion

This study demonstrated that predator risk treatment significantly affected the development and fecundity of *B. dorsalis*, although it did not have a significant effect on body weight at death. *B. dorsalis* subjected to caged-predator and predator-odor treatments exhibited shorter developmental times and higher fecundity. These findings suggest a potential correlation between the predation risk imposed by *H. patellifera* and developmental changes in *B. dorsalis*. Moreover, they indicate that predator odors alone can induce a predation risk effect on the development of *B. dorsalis* similar to that induced by a real predator.

Studies have shown that under predation risk, prey face a trade-off between responding to predation threats and lifespan [49,50]. For instance, as predation pressure increases, the developmental time of the larvae of *Tyrophagus putrescentiae* extends, while the adult lifespan shortens [48]. In our study, we observed that the developmental time of *B. dorsalis* decreased following exposure to caged-predator risk. Similarly, exposure to predator odors also reduced the developmental time of *B. dorsalis* compared to the control, suggesting that when faced with predation risks, *B. dorsalis* might allocate more resources and energy to defense rather than to development, consequently altering their developmental time. This finding is consistent with previous research where, under the risk of predation by bats, *Spodoptera litura* exhibited a reduced lifespan [51]. This trade-off may have an important impact on the fitness of the threatened individual and may then be amplified to affect the population, which suggests an indirect effect of predators on pest population dynamics [9,52,53,54].

Furthermore, we discovered that the impact of predation risk on prey *B. dorsalis* development time was gender-specific; female *B. dorsalis* treated with caged predator had a shorter developmental time than those in the control treatment, while this was not observed in males. This disparity was also found in the phytophagous mite (*Tyrophagus putrescentiae*), where, regardless of being subjected to predator stress throughout the life cycle or at different life stages, the lifespan of males was significantly longer than that of females [22]. The variation in developmental time responses between females and males could be attributed to the fact that females bear the responsibility for fecundity and need to allocate most of their resources to reproduction, leaving less for development under predation risks [8,48,55,56].

A trade-off between predation risks and reproduction has also been indicated by many studies [25,48,51]. For example, predation-stressed phytophagous mites (*Tyrophagus putrescentiae*) were found to reduce fecundity and allocate resources to anti-predation [22], which is contrary to our observations. The fact that fecundity increased under exposure to predation risks (both caged-predator and predator-odor treatments) was observed. The difference may be due to differences in the prey species, predator type, hunting model, and strength of predation risks, and prey may adjust their reproduction accordingly [57,58].

However, the developmental time of female *B. dorsalis* treated with predation risk decreased, but the fecundity was higher than that of the control treatment. A similar situation has also been observed where, when threatened by long-term predation, the lifespan of the aphid *Rhopalosiphum padi* will decrease, but the fecundity rate will increase [59]. The increased number of offspring may allow the individual that is eaten by a predator to be compensated [60]. This suggests that in our studies, the resources or energy diverted from development by female *B. dorsalis* may be allocated to fecundity or anti-predation, or to both of them. The increased number of offspring may result in a satiety effect in the predator [61], allowing enough individuals to survive to establish a new population. Thus, the indirect predation risk effects from *H. patellifera* have a major impact on the population dynamics of *B. dorsalis*.

Our study showed that there was no significant difference in body weight at death between the control and caged-predator or predator-odor treatments. This differs from the results of previous studies. Under predation stress, the development of *Helicoverpa armigera* was accelerated, and the adult size became larger [62]. Studies also indicated that prey tended to become larger when facing a predation risk [63]. It is still unknown why body weight is not shown to be affected in our present results; however, we did observe hesitation in *B. dorsalis* to approach and feed on the food in the experimental cage under predation risk. As food is an important factor influencing body weight, this behavior should reduce its body weight. A probable reason is that the stressed *B. dorsalis* individual may increase the quantity and/or quality of its food (for example, eat more or increase the food conversion rate), thus suffering fewer effects on its body weight.

To conclude, this study has shown that predation risks from the predatory mantis *H. patellifera* can shorten the developmental time and increase the fecundity of *B. dorsalis*. This may represent a resource or energy trade-off between development and predator avoidance, which could have a profound impact on the population dynamics of *B. dorsalis*. Studies have shown that indirect predation risk may significantly impact the development and reproduction of *B. dorsalis*, akin to the effects of direct predation. This insight may alter our understanding of the use of *H. patellifera* in biological control, suggesting that the indirect predation risk effect should be considered. Importantly, we found that the odor of predators alone can induce a risk response similar to that of actual predators, indicating the potential for a novel pest-control method—utilizing the odor of predators as a means to control pests, rather than directly using the predators. This approach may be more economical and more easily implemented. However, it is first necessary to identify the nature of the predator odor before applying this odor-based strategy to actual pest control.

## Data Availability

The data presented in this study are available on request from the corresponding author.

## Appendix A

**Table A1:** The COX proportional hazards model examined the effects of predation risk treatments, sexes, and their interaction on the survival of *B. dorsalis* under caged predator (a) and predator odors experiments (b). The bold value is the significant value.

Response Variables	Predictor Variables	Coef	Exp (coef)	Se (coef)	Z	Pr (>|z|)
(a) Developmental time (caged predator experiments)	treatments	0.349	1.417	0.164	2.132	**0.033**
	sexes	−0.107	0.898	0.163	−0.656	0.512
	Treatments: sexes	−0.346	0.708	0.226	−1.529	0.126
(b) Developmental time (predator odors experiments)	treatments	0.395	1.485	0.172	2.304	**0.021**
	sexes	−0.025	0.975	0.172	−0.145	0.885

**Table A2:** The ANOVA analysis examined the effects of predation risk treatments, fecundity time, and their interaction on the fecundity of *B. dorsalis* under caged predator (a) and predator odors experiments (b). The bold value is the significant value.

Response Variables	Predictor Variables	df	chisq	Pr (>F)
(a) Fecundity (caged predator experiments)	treatment	1	33.10	**<0.001**
	time	1	83.48	**<0.001**
	treatment:time	1	8.33	**0.004**
(b) Fecundity (predator odors experiments)	treatment	1	3.95	**<0.001**
	time	1	359.95	**0.047**
	treatment:time	1	1.76	0.183

**Table A3:** The linear mixed model was used to analyze the impact of predation risk, sexes and time on the body weight of *B. dorsalis* at death under caged predator (a) and predator odors experiments (b). The bold value is the significant value.

Response Variables	Predictor Variables	df	chisq	Pr (>F)
(a) Body weight at death (caged predator experiments)	time	1	2.599	0.107
	treatment	1	1.214	0.271
	sex	1	13.437	**<0.001**
	time:treatment	1	0.171	0.679
	time:sex	1	0.818	0.366
	treatment:sex	1	0.487	0.485
	time:treatment:sex	1	0.471	0.493
(b) Body weight at death (predator odors experiments)	time	1	19.753	**<0.001**
	treatment	1	2.012	0.156
	sex	1	9.781	**0.002**
	time:treatment	1	1.440	0.230
	time:sex	1	1.313	0.252
	treatment:sex	1	0.280	0.597
	time:treatment:sex	1	1.574	0.210
